# Antimicrobial Compounds from Microorganisms

**DOI:** 10.3390/antibiotics11030285

**Published:** 2022-02-22

**Authors:** Cynthia Amaning Danquah, Prince Amankwah Baffour Minkah, Isaiah Osei Duah Junior, Kofi Bonsu Amankwah, Samuel Owusu Somuah

**Affiliations:** 1 Department of Pharmacology, Faculty of Pharmacy and Pharmaceutical Sciences, College of Health Sciences, Kwame Nkrumah University of Science and Technology, PMB, Kumasi, Ghana; princederoyal@gmail.com; 2 Global Health and Infectious Disease Research Group, Kumasi Centre for Collaborative Research in Tropical Medicine, College of Health Sciences, Kwame Nkrumah University of Science and Technology, PMB, Kumasi, Ghana; 3 Department of Optometry and Visual Science, College of Science, Kwame Nkrumah University of Science and Technology, PMB, Kumasi, Ghana; oseiduahisaiah@gmail.com; 4 Department of Biomedical Sciences, University of Cape Coast, PMB, Cape Coast, Ghana; kamankwah@stu.ucc.edu.gh; 5 Department of Pharmacy Practice, School of Pharmacy, University of Health and Allied Sciences, PMB, Ho, Ghana; sosomuah@uhas.edu.gh

**Keywords:** antimicrobial peptides, secondary metabolites, natural products, microorganisms, drug discovery, model organisms, omics-informed drug discovery, structure-activity

## Abstract

Antimicrobial resistance is an exigent public health concern owing to the emergence of novel strains of human resistant pathogens and the concurrent rise in multi-drug resistance. An influx of new antimicrobials is urgently required to improve the treatment outcomes of infectious diseases and save lives. Plant metabolites and bioactive compounds from chemical synthesis have found their efficacy to be dwindling, despite some of them being developed as drugs and used to treat human infections for several decades. Microorganisms are considered untapped reservoirs for promising biomolecules with varying structural and functional antimicrobial activity. The advent of cost-effective and convenient model organisms, state-of-the-art molecular biology, omics technology, and machine learning has enhanced the bioprospecting of novel antimicrobial drugs and the identification of new drug targets. This review summarizes antimicrobial compounds isolated from microorganisms and reports on the modern tools and strategies for exploiting promising antimicrobial drug candidates. The investigation identified a plethora of novel compounds from microbial sources with excellent antimicrobial activity against disease-causing human pathogens. Researchers could maximize the use of novel model systems and advanced biomolecular and computational tools in exploiting lead antimicrobials, consequently ameliorating antimicrobial resistance.

## 1. Introduction

The surge in antimicrobial resistant infections and the concurrent increase in multidrug resistant organisms has jeopardized the healthcare system and threatens public health. Annually, thousands of lives are lost due to resistant infections, and without robust systems, the world would experience over 10 million yearly deaths [1]. Currently, the growing antimicrobial resistance has rendered the efficacy of antimicrobials of questionable utility [2]. Therefore, the search for alternate antimicrobial agents has become a necessity. 

Over the past decade, natural products have been heavily relied upon as sources of therapeutic agents, with antimicrobials being one of the most compelling biomolecules. In particular, they constitute more than two-thirds of newly approved medicinal products used for pharmaceutical applications [3]. Unlike microbial-originated antibiotics, plant-based antimicrobials have been extensively explored and with varied applications in medicine, veterinary, agriculture, and biotechnology. Microorganisms are recognized as producers of bioactive compounds with antibacterial, antifungal, and cytotoxic bioactivity [4,5,6,7]. Again, their production of functionally rich secondary metabolites enables them to thrive in varied environmental conditions. Researchers have recently paid attention to microbes as untapped reservoirs for novel antimicrobial agents due to their distinctive biological properties [8]. Specifically, the invention of state-of-the-art molecular biology, genetic, genomic, and computational tools have facilitated the mining of microbial structural systems to enhance drug discovery [9,10,11].

**Figure 1:**
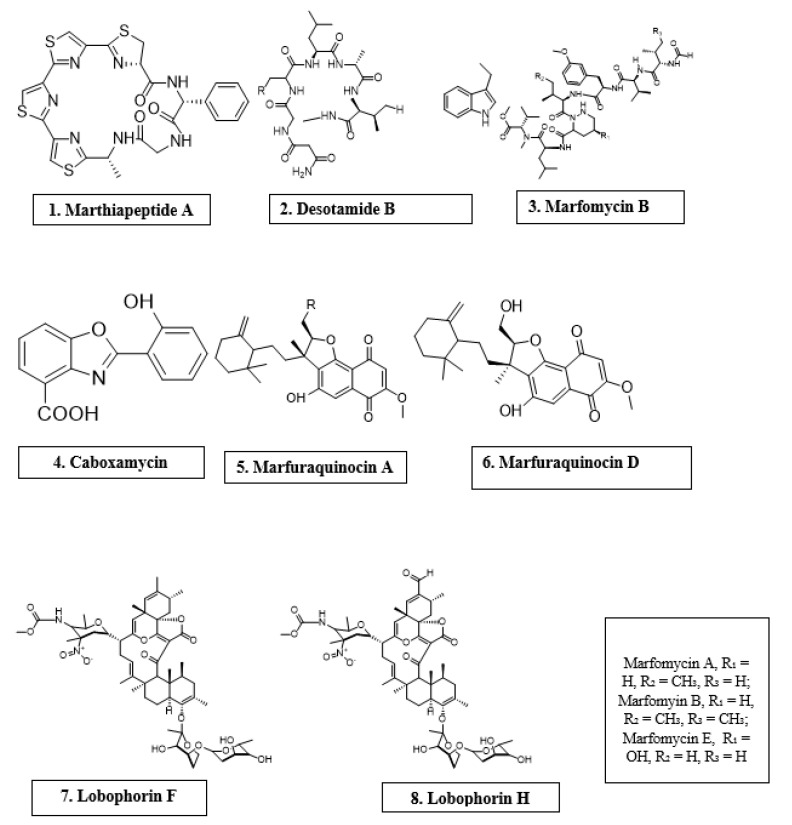
Antimicrobial compounds of different classes isolated from microorganisms.

Microorganisms are biotic, ubiquitous, diverse creatures broadly categorized into viruses, bacteria, archaea, fungi, and protists. Predominantly, bacteria and fungi are explored as potential sources of novel antimicrobial agents. For instance, cyclic peptides- mathiapeptide A, destotamide B, Marfomycins A, B, E; spirotetronates polyketides-abyssomycin C, Lobophorin F, H, as well as alkaloids and sesquiterpenes derivatives, caboxamyxin and mafuraquinocins A, D (Table 1; Figure 1) isolated from bacteria, have antimicrobial properties suicidal against clinically resistant bacteria, including *Staphylococcus aureus* (*S. aureus*), Methicillin-resistant *Staphylococcus aureus* (*MRSA*), *Micrococcus luteus* (*M. luteus*), *Bacillus subtilis* (*B. subtilis*), and *Enterococcus faecalis* (*E. faecalis*) [12]. Similarly, ambuic acid analogs, the penicyclones classes; depsidone analogs, the spitomastixones groups; xanthones derivatives, emerixanthones, and engyodontiumsones from fungi, exhibit an anti-infective activity against Gram-negative bacteria, *Escherichia coli* (*E. coli*) and *Klebsiella pneumoniae* (*K. pneumoniae*), and several other Gram-positive pathogenic bacteria [12]. Furthermore, in vivo and in vitro assays have also demonstrated the anti-infective potentials of other microbial products extracted from cyanobacteria [13,14], microalgae [14,15], and yeast [16,17].

The recent technological advances have primed scientists to produce synthetic antimicrobials through chemical and structural modification of natural products to overcome antibiotic resistance. In particular, component-based synthesis, structured-guided designs, and X-ray crystallography have enabled the fabrication and visualization of novel antimicrobials from primogenitor cell lines [18]. A typified example is oxepanoprolinamide, a derivative of lincosamide [18], which showed a greater propensity to overcome ATP binding cassette (ABC) F-, emerging erm B (Erm-), and Cfr gene-multidrug resistance, and with increased therapeutic effect against resistant bacterial strains [18]. Given the emergence of diverse strains of resistant microorganisms and the advent of modern tools, new evidence is warranted to enhance bioprospecting of new antimicrobials. Therefore, the overarching goal of this review is to report on novel antimicrobial compounds from microorganisms and further explore the contemporary tools used in antimicrobial drug discovery.

## 2. Bacterial Sources of Antimicrobials

Lactic acid bacteria (LAB) have the tendency to produce antimicrobial compounds (i.e., bacteriocin, organic acids, diacetyl, and hydrogen peroxide), which are effective against harmful bacteria [19]. Bacteriocin production by *Lactobacillus pentosus* (*L. pentosus*) ST712BZ isolated from boza antagonizes the proliferation of *Lactobacillus casei* (*L. casei*), *E. coli*, *Pseudomonas aeruginosa* (*P. aeruginosa*), *E. faecalis*, *K. pneumoniae, and Lactobacillus curvatus* (*L. curvatus*) [20]. Bacteriocins are low molecular weight polypeptides synthesized in ribosomes and comprise 20–60 amino acid residues [19]. In 1925, Andre Gratias discovered bacteriocin when he realized that the growth of some *E. coli* strains was being impeded by an antibacterial compound, which he named colicin V [21]. Although there are different classes of bacteriocins produced by other Gram-positive and Gram-negative bacteria as well as archaea, those produced by LAB are the most studied due to their use as food preservatives as well as the frequent incidence of food-borne infectious diseases [21]. According to Klaenhammer, four groups of bacteriocins exist based on their molecular mass, enzyme sensitivity, thermos-stability, presence of post-translationally modified amino acids, and mode of action [22]. Class I is made up of lantibiotics and can further be grouped into Ia or Ib depending on the structure and charge of compound. Class II bacteriocins consist of heat-stable peptides with molecular masses less than 10 kDa and can also further be categorized into classes IIa, IIb, and two other types of IIc [22]. The third class, which consists of high molecular weight (usually >30 kDa) thermo-labile peptides, are represented by Helveticin J and the last class IV, comprises a mixture of large peptides and carbohydrates or lipids [23]. However, since there is no standard classification for bacteriocins, studies by Cotter et al. [24], Drider et al. [25], and others reveal contrasting theories about their classification. In modern times, classification of bacteriocins into three classes based on genetics and biochemical properties is most often used. These classes are class I (lantibiotics), class II (non-lantibiotics), and class III [25]. Each class of bacteriocins has their own way of exhibiting antimicrobial activity based on their primary structure [26] see Table 2. Some bacteriocins attack energized membrane vesicles of target microbes by tampering with their proton motive force [27], while others enter the cell and disrupt gene expression and protein synthesis [26]. Lantibiotics fight bacteria in two ways. They alter the bacterial cell wall formation process by binding to lipid II, a hydrophobic carrier of peptidoglycan monomers from the cytoplasm to the cell wall, making the cell unsuitable for certain actions. Lipid II is responsible for membrane insertion and pore formation in the cell membrane of bacteria [26,28,29].

Non-lantibiotics on the other hand, kill their target cells by binding to MptC and MptD subunits of mannose phosphotransferase permease (Man-PTS) causing an intra-membrane channel to open and ions to continuously diffuse through [29,30]. Without requiring any receptor molecule circular bacteriocins owing to their high net positive charges are electrostatically attracted to the negatively charged bacteria membrane. This interaction leads to pore formation, efflux of ions, changes in membrane potential, and eventually cell death [31].

Bacteriolysins enhance cell wall hydrolysis causing the cell to gradually break down [32,33]. Non-bacteriolysins disrupt glucose uptake in target cells, consequently starving them to death [34,35,36]. Interactions between antimicrobial compounds and their susceptible microbes can be synergistic or antagonistic [37]

In veterinary medicine, bacteriocins, such as nisin, have been clinically used to prevent dentobacterial plaque and gingivitis in dogs [38,39], as a result of its brutal action against strains of *E. faecalis* and other canine periodontal disease-causing bacteria [26].

Rhamnolipid are popular anionic biosurfactants, generally produced by some species of *Pseudomonas* and *Burkhloderia* [40,41] These compounds have shown a broad spectrum of biological activities, including activities against microorganisms, biofilm, tumors, and oxidation [42,43,44]. Of great interest is their activity against *Herpes simplex* virus 1 and 2 (HSV-1 and HSV-2) and bovine coronaviruses, via interactions with viral lipid membranes and thereby altering viral membrane glycoproteins [45,46]. Rhamnolipids (M15RL) produced by the Antarctic bacterium, *Pseudomanas gessardii* (*P.gessardii*) M15, has recently been reported to exert high antiviral activity against Coronaviridae and Herpesviridae families, especially against severe acute respiratory syndrome coronavirus 2 (SARS-CoV-2) [47]. 

**Table 2:** Examples of bacteriocins, organisms that produce them and microbes that are susceptible to them.

Bacteriocin	Producer of Bacteriocin	Susceptible Microorganisms	Reference(s)
Nisin A	*Lactococcus lactic subsp. lactis*	*E. faecalis* ssp. *Liquefaciens*, *Streptococcus equinus*, *Staphylococcus epidermidis* (*S. epidermidis*), *S. aureus*, *Streptococcus uberis* (*S. uberis*), *Streptococcus dysgalactiae* (*S. dysgalactiae*), *Streptococcus agalactiae* (*S. agalactiae*), *Streptococcus suis* (*S. suis*) *Mycobacterium avium subsp. Paratuberculosis*	[48,49,50]
Nisin ANisin V	*L. lactis* NZ9700*L. lactis* NZ9800nisA:M21V	*Listeria monocytogenes*	[51]
Pediocin A	*Pediococcus pentosaceus* FBB61	*Clostridium perfringens*	[52]
Enterocin M	*Enterococcus faecium* AL41	*Campylobacter* spp., *Clostridium* spp.	[53]
Enterocin CLE34	*Enterococcus faecium* CLE34	*Salmonella pullorum*	[26,54]
Enterocin E-760	*Enterococcus durans*, *Enterococcus faecium*, *Enterococcus hirae*	*Salmonella enterica serovar Enteritidis*, *S. enterica serovar Choleraesuis*, *S. enterica serovar Typhimurium*, *S. enterica serovar Gallinarum*, *E. coli O157:H7*, *Yersinia enterocolitica*, *S. aureus*, *Campylobacter jejuni*	[55]
Lacticin 3147	*Lactococcus lactis* DPC3147.	*S. dysgalactiae*, *S. agalactiae*, *S. aureus*, *S. uberis*, *Mycobacterium avium subsp. paratuberculosis*	[50,56]
Macedocin ST91KM	*Streptococcus gallolyticus subsp.macedonicus* ST91KM	*S. agalactiae*, *S. dysgalactiae*, *S. uberis*, *S. aureus*	[57]

## 3. Bacterial Sources of Antifungal Compounds

Red pigmented pradimicins A, B, and C are products of the bacteria *Actinomadura hibisca* (*A. hibisca*) [58]. These pradimicins exhibit antifungal properties against *Candida* and *Aspergillus species* as well as other fungi [59] see Table 3. Spectral analysis and chemical degradation reveals pradimicins structurally to be a benzo[α]napthacenequinone carrying D alanine and sugars [58]. Pradimicins use specific binding recognition to bind to terminal D mannosides of the cell wall of susceptible microbes to form a D-mannoside, pradimicin, and calcium complex that destroys fungal cell membrane [59].

*Actinoplanes species* also produce antifungal metabolites. An example is *Actinoplanes ianthinogenes* (*A. ianthinogenes*), which produces purpuromycin, a compound that has activity against *Trycophyton mentagrophytes* (*T. mentagrophytes*) [60]. Another species is known as *Octamycini* produces octamycin [60]

Soil-occurring *Micromonospora species* have been identified with the production of antifungal compounds [60]. *Micromonospora species* ATCC 53803, through metabolism, produces spartanamycin B as a secondary metabolite, which has activity against *Candida albicans* (*C. albicans*), *Aspergillus cladosporium* (*A. Cladosporium*), and *Cryptococcus* spp. *Micromonospora neiheumicin* (*M. neiheumicin*) produces neihumicin, which is active against *Saccharomyces cerevisae* (*S. cerevisiae*) activity [61]. Sch 37137, a dipeptide formed by *Micromonospora species* SCC 1792, also fights against dermatophytes and *Candida species* [62]. Lastly, Nishizawa et al. reported that *Micromonospora species* SF-1917 produces nucleoside antibiotics, dapiramicins A and B. Dapiramicins B inhibits growth of *Rhizoctonia solania* (*R. solania*) of rice plants in a greenhouse test [63].

Aerobic Gram-positive branching bacilli, *Streptomyces species*, yield some antifungal compounds. These compounds include nystatin, phoslatomycins [64], UK-2A, B, C, D [65], phthoxazolin A [66], faeriefungin [67], butyrolactols A and B [68], sultriecin [69], polyoxin [70]), and dunaimycins [71].

Some bacilli species are also known to be the source of several antifungal compounds. *Bacillus subtilis* produces iturin and other closely related peptides, including bacillomycin D, F, and L, mycosubtilin, and mojavensin. These agents have been shown to be active against phytopathogens and hence, have been commercialized as biological control agents against fungal plant pathogens. Notably, there has not been any reported resistance against fungi for these compounds. These agents act by creating pores in the membrane of susceptible fungi, thereby causing leakage of cell contents and subsequent cell death [60,72].

According to Kerr, the compounds; azoxybacilin, bacereutin, cispentacin, and mycocerein can be isolated from the products of *Bacillus cereus* (*B. cereus*) and are active against *Aspergillus species*, *Saccharomyces* spp., *Candida albicans*, and other fungi. [60] Another *Bacilli species*, *B. licheniformis*, produces fungicin M-4 and peptide A12-C [73,74]

The compound, pyrrolnitrin, has been reported by Chernin et al. to be the factor responsible for the antimicrobial action of *Enterobacter agglomerans* (*E. agglomerans*) on the *Candida species*, *Aspergillus niger* (*A. niger*), dermatophytes and phytopathogenic fungi. *Enterobacter agglomerans* again produces herbicolins A and B. which are active against yeasts and filamentous fungi [75,76,77]. CB-25-1, a water soluble dipeptide, produced by *Serratia plymuthica* (*S. plymuthica*) is known to inhibit growth of *C. albicans* [78].

*P. aeruginosa* present in the gut of a normal person has been identified as the source of three antifungal compounds, namely dihydroaeruginoic acid [79], pyocyanin, and 1-hydroxyphenazine [80]. Other antimicrobial compounds produced by pseudomonas include 2,4-diacetophluoroglucinol [81], peptide pseudomycin family [82], caryoynencins [83], and cyclic hydroxamic acid, G1549 [84].

*Burkholderia species* are another bacterial source of antimicrobial compounds. Cepacidine A, which antagonizes plant and animal fungi growth, can be generated by *B. cepacia* [85]. *B. cepacia* also produces cepalycin [86], xylocandins [87], and heptylmethyl-quinolinone [88]. Another group of antibacterial compounds is enacyloxcins, known to originate from the Burkholderia species [89]. Enacyloxcins consists of eight closely related antibacterial compounds (86–87). Maltophilin is the active compound responsible for the antifungal action of the Rhizosphere strain of *Stenotrophomonas maltophilia.* Polyenic antibiotics produced by the genus *Gluconobacter* have also been reported to possess some antifungal activity against the fungus *Neurospora crassa* but not against yeast [90]

## 4. Fungal Sources of Antimicrobials

The discovery of penicillin G in 1928 from fungal species has led to the exploration of these organisms [91]. Their ability to produce a plethora of active secondary metabolites that can serve as lead compounds for the synthesis of antimicrobials is significant.

*Hormonema species* that yielded enfumafungin, a triterpenoid, was discovered over a decade ago and was shown to be highly effective against *Candida* spp. and *Aspergillus* spp. It is still being studied in order to produce a number of developmental compounds [92]. Enfumafungin yielded a semisynthetic derivative, SCY-078 that is in phase II clinical trial. The biosynthetic encoding genes for this peculiar triterpenoid were only recently discovered, but have shown a lineage of hopene-type cyclases, including ERG7, which is necessary for the biosynthesis of fungal ergosterol [93], see Table 4.

Testing of metabolites in the strobilurins, known as antifungal agents in agriculture, has not been explored since it was identified in 1999 as being harmful to humans [94]. In recent times however, favolon, produced by *Favolaschia calocera* (*F. calocera*), a metabolite of strobilurins has been identified and has been shown to be less toxic but with potent antifungal activity against human pathogens [95].

Fungal metabolites, by their ability to interfere with quorum sensing, inhibits the formation of biofilms. Coprinuslactone, derived from *Coprinus comatus* (*C. comatus*), acts on *P. aeruginosa* biofilms [96]. Microporenic acid A from a Kenyan basidiomycete also inhibits *S. aureus* and *C. albicans* biofilms and has an added advantage of destroying pre-formed biofilms [97]. Biofilm inhibitors are promising adjuncts to antibiotics.

Mutulins and its derivative, retapamulin from the basidiomycete *Clitopilus passeckerianus*, represents a new area in search of antimicrobials. They have been shown to have potent antibacterial activity, and more derivatives are undergoing clinical trials. The drawback with them is the difficulty in reaching a large scale since they grow slowly and generate low yields [96].

A novel rubrolide, rubrolide S, discovered from the marine fungus *Aspergillus terreus* (*A. terreus*) OUCMDZ-1925, has been shown to significantly inhibit the activity of *Influenza A virus* (H1N1) [98]. A novel hybrid polyketide, Cladosin C, isolated from *Cladosporium sphaerospermum* 2005-01-E3, has also shown activity against *Influenza A* H1N1 [99]. *Penicillium chrysogenum* PJX-17 has also shown to be the source of two antiviral sorbicillinoids, named sorbicatechols A and B, with significant activity against the H1N1 [100].

Trypilepyrazinol and β-hydroxyergosta-8,14,24 (28)-trien-7-one isolated from extracts of the fungus *Penicillium* sp. IMB17-046 has shown broad spectrum antiviral activities against different types of viruses, including human immunodeficiency virus (HIV) and hepatitis C virus (HCV) [101]. *Aspergillus niger* SCSIO Jcsw6F30 produces aspernigrin C and malformin C, which exhibited significant antiviral activity against HIV-1 [102]. Antimycin A, an isolate from *Streptomyces kaviengensis* (F7E2f), has shown strong activity against Western equine encephalitis virus (WEEV) via the interruption of mitochondrial electron transport and pyrimidine biosynthesis [103].

**Table 4:** Antimicrobial activity of chemical compounds from fungi.

Microorganism	Compounds	Antimicrobial Activity	Reference(s)
*Hormonema* spp.	Enfumafungin	*Candida* spp. and *Aspergillus* spp.	[92]
*F. calocera*	Favolon	*Candida tenuis* and *Mucor plumbeus*	[95]
*C. comatus*	Coprinuslactone	*P. aeruginosa*	[96]
*Sanghuangporus* spp.	Microporenic acid A	*S. aureus* and *C. albicans*	[97]
*Aspergillus terreus*	Rubrolide S	*Influenza A virus* (*H1N1*)	[98]
*Cladosporium sphaerospermum 2005-01-E3*	Cladosin C	*Influenza A H1N1*	[99]
*Penicillium sp. IMB17-046*	Trypilepyrazinol and *β*-hydroxyergosta-8,14,24 (28)-trien-7-one	*HIV and HCV*	[101]

## 5. Antimicrobial Peptides

Antimicrobial peptides (AMPs) are a diverse class of naturally occurring molecules that are derived from various microorganisms, such as bacteria, fungi, parasites, and viruses that act as host defense for these microorganisms [104]. AMPs are small-sized peptides and consist of large numbers of lysine or arginine residues and hence, mostly cationic. This positive nature enables AMPs to interact with microbial membranes that are largely negatively charged. Some AMPs, however, are anionic in nature [105]. A total of 3791 AMPs has been reported from various microorganisms [106]. For a long time, treatment of infectious diseases relied heavily on antibiotics, and rightly so, before the issue of antimicrobial resistance (AMR). Recently, AMPs have received significant audience and have shown excellent antibacterial activity against pathogenic organisms by acting on multiple targets on the plasma membrane and intracellular targets; they have broad spectrum activity and low tendency to induce resistance, high efficacy at low concentrations, and synergistic action with conventional antibiotics, serving as a suitable alternative to the traditional antimicrobials [107,108]. AMPs have shown antibacterial activity, antifungal activity, antiviral activity, antiparasitic activity, and immunomodulatory activity [104].

AMPs have a wide inhibitory effect on common pathogens, such as VRE, *Acinetobacter baumannii*, and MRSA in clinical medicine, and *S. aureus*, *Listeria monocytogenes*, and *E. coli* in food and *Salmonella*, *Vibrio parahaemolyticus* in aquatic products. AMPs, such as nisin, cecropins, and defensins, have shown excellent activity against Gram-positive and Gram-negative bacteria. AMPs P5 (YIRKIRRFFKKLKKILKK-NH_2_) and P9 (SYERKINRHFKTLKKNLKKK-NH_2_), which are designed based on *Aristicluthys nobilia* interferon-I, have been shown to inhibit MRSA [109].

The need for AMPs has seen a significant surge due to AMR and can be employed in human health and agriculture. While applications of AMPs are diverse, and calls for large-scale production are being made, synthesis of AMPs is low and susceptible to proteolytic degradation due to the L-amino acids in them [107]. Genetic engineering is one important strategy being employed to increase yield of AMPs [110]. The use of chloroplast engineering, heterologous expression of AMPs, transgenic expression of AMPs in plants, and the application of gene-editing tools and technologies provide scope for future research [111].

## 6. Antiviral Peptides

Viral infections have been reported since ancient times. It was only in the 19th century that scientists were able to isolate viruses. Since then, substantial investigation regarding the control of viral reproduction and infection in humans, such as the smallpox eradication some years ago, has been carried out [112]. Viruses remain as one of the major causes of human disease and this may be due to difficulty in discovery and the time consumed in the development of new vaccines [112]. Antiviral drugs are being employed; however, there are side effects associated with their usage. Some antivirals also tend to have low efficacies due to reports of viral resistance and also due to the emergence and re-emergence of viral epidemics in relatively short periods of time, as observed in H1N1, Ebola, and Zika viruses. The demand for production of new antiviral drugs with higher efficacy is on the rise. Recent studies have highlighted the antiviral proteinaceous compound, antiviral peptides (AVPs), as a defensive barrier [112]. Antiviral peptides destroy viruses chiefly by inhibiting virus attachment and virus cell membrane fusion, destroying the virus envelope, or inhibiting virus replication [113].

Clavanin is an example of an AVP derived from a tunicate called *Styela clava* [114]. Clavanin A has been tested against rotavirus and adenovirus [115], while clavanin B has shown inhibitory activity against HIV [116]. Anti-HIV peptides, such as defensins (i.e., α- and β-defensins), LL-37, gramicidin D, carin 1, maximin 3, magainin 2, dermaseptin-S1, dermaseptin-S4, siamycin-I, Siamycin-II, and RP 71955, and antiviral peptide enfuvirtiude, have been commercialized as medications for management of HIV [113].

Due to the COVID-19 pandemic, antiviral peptides are being produced against the coronavirus. The lipopeptide, EK1C4, derived from EK1 (SLDQINVTFLDLEYEMKKLEEAIKKLEESYIDLKEL), has been shown to be the most effective fusion inhibitor against COVID-19 S protein-mediated membrane fusion [117]. Moreover, research has demonstrated that AMP Epi-1 facilitates the inactivation of virus particles and is effective against the foot-and-mouth disease virus [113].

Since the majority of viral infections still have no available treatment, novel antiviral molecules are indispensable and antiviral peptides may present a new phase in the search of these molecules. The potential problems, such as the cost of production and poor oral absorption of these compounds, needs to be addressed to ensure AVPs reach the clinical trial phase.

## 7. Other Microbial Sources of Antimicrobial Compounds

Organisms, such as algae, bryozoans, corals, molluscs, sponges, tunicates, and viruses, are considered potential sources of novel antimicrobials [118,119,120,121,122,123,124,125,126,127] as seen in Table 5. Their external body structures could serve as an avenue for new bioactive compounds. Additionally, the internal enzymatic machinery of some of these microorganisms enables them to produce secondary metabolites with antimicrobial properties. For example, *Pseudovibrios species*, a marine bacterium of the order Rhodobacterales and class alphaproteobacteria, has bioactive structural composition [124,128] coupled with harbored polyketide synthases, non-ribosomal peptide synthases, or hybrid enzyme systems that putatively aid them to produce secondary metabolites and new bioactive compounds with antimicrobial activity against varying clinical strains, notably *Staphylococcus aureus*, *Bacillus subtilis*, and *Escherichia coli* [129,130]. Psychrophiles, extremophilic organisms that tolerate very low temperatures, were also investigated as a source of new antimicrobials. Given the varying environmental conditions between psychrophiles and temperate regional dwellers and their adaptive evolution, the bioactive compounds produced by the former might presumably differ from the latter, and that merits its consideration as an antimicrobial source. Tadesse and colleagues identified Synoxazolidinones A and B, oxazolidinone derivative antimicrobial isolates from the Norwegian sea squirt, which showed antibacterial activity against methicillin-resistant *Staphylococcus aureus* (MRSA) [126]. Sanchez et al. also reported the bacteriocin properties of Serraticin A, a bioactive compound produced by *Serratia proteomaculans* and with antimicrobial activity against *Escherichia coli* and *Salmonella enterica*. This compound is putatively considered to exhibit such activity by inhibiting deoxyribonucleic acid (DNA) synthesis [131]. Similarly, Phelan and colleagues found subtilomycin, a lantibiotic from the marine sponge *Haliclona simulans*, known to exhibit polymyxin B activity (cell membrane inhibition or pore formation) against strains of *Bacillus cereus*, *Bacillus megaterium*, *Clostridium sporogenes*, *Listeria monocytogenes*, *Listeria innocua*, *Staphylococcus aureus*, *MRSA*, and vancomycin-resistant *Staphylococcus aureus* [122,125]. Lobophorin, a spirotetronate antibiotic from seaweed sediments, exhibited activity against bacteria and fungi. In particular, lobophorins display their antibacterial effect by inhibiting tetrahydrofolate synthesis [118,132]. Kim et al. demonstrated the antibacterial activity of isolated bioactive compounds from the artic lichen. Specifically, the antimicrobials had action against Gram-positives, *Staphylococcus aureus*, *Bacillus subtilis*, *Micrococcus luteus*, Gram-negatives, *Pseudomonas aeruginosa*, *Escherichia coli*, *and Enterobacter cloacae* [133]. Arctic bryozoans in the same vein harbored eusynstylamides with antibacterial activity against *Pseudomonas aeruginosa*, *Staphylococcus aureus*, *Escherichia coli*, and *Corynebacterium glutamicum* [119,121]. Concurrently, in a study to characterize the antibacterial and antifungal activity of Antarctic psychrophiles, Giudice et al. retrieved and screened 580 bacteria isolates from two phylogenic sources (actinobacteria and gamma proteobacteria) against terrestrial microorganisms, mainly Gram bacteria and eukaryotic yeast [134]. Overall, 22 of them showed varying degrees of antibacterial activity against *Bacillus subtilis*, *Escherichia coli*, *Micrococcus luteus*, and *Proteus Mirabillis* [134]. Similarly, 132 bacteria isolates of the genera Antrobacter, Pseudoalteromas, Psychrobacter, Shewanella, and Roseobacter retrieved from the Antartic sponges *Anoxycalyx joubini*, *Haliclonissa verrucosa*, and *Lissodendoryx nobilis*, and screened against opportunistic bacteria pathogens exhibited a bacteriostatic action against the *Burkholderia cepacia* bacteria complex [135]. The *Nocardioides species*, a halophilic microbe from Antarctic soil, produced antimicrobial compounds that demonstrated activity against Gram-positive and Gram-negative bacteria and with the greatest effect on *Staphylococcus aureus* and *Xanthomonas oryzae* [136]. While viruses traditionally might seem to pose a threat to humanity, the mining of their protein constituents has revealed their antimicrobial propensity. An exploration of the antimicrobial peptides (AMP) and cell-penetrating properties of viral proteins by Miguel Frere and colleagues identified capsid proteins from encapsulated and non-encapsulated viruses with thousands of AMP amino acid sequences, conferring it an antimicrobial activity [123]. 

**Table 5:** Other sources of antimicrobial compounds.

Microbial Sources	Compound(s)	Susceptible Organism(s)	Reference(s)
*Synoicum pulmonaria*	Synoxazolidinones A and B	MRSA	[126]
*Serratia proteomaculan*	Serraticin A	*Escherichia coli* and *Salmonella enterica*	[131]
*Haliclona simulans*	Subtilomycin	*Bacillus cereus*, *Bacillus megaterium*, *Clostridium sporogenes*, *Listeria monocytogenes*, *Listeria innocua*, *Staphylococcus aureus*, *MRSA*, and Vancomycin-resistant *Staphylococcus aureus*	[122,125]
*Ochrolechia* spp.	PAMC26625	Gram-positives: *Staphylococcus aureus*, *Bacillus subtilis*, *Micrococcus luteus;* Gram-negatives, *Pseudomonas aeruginosa*, *Escherichia coli*, and *Enterobacter cloacae*	[133]
*Tegella cf. spitzbergensis*	Eusynstylamides	*Pseudomonas aeruginosa*, *Staphylococcus aureus*, *Escherichia coli*, and *Corynebacterium glutamicum*	[119,121]
*Nocardioides* spp.	Strain A-1	*Staphylococcus aureus* and *Xanthomonas oryzae*	[136]

## 8. Tools and Techniques Used for Antimicrobial Drug Discovery from Microorganisms

The growing antimicrobial resistance merits the search for new bioactive compounds with activity against disease-causing pathogens. Yet the effort towards achieving this feat has been hindered over the years, owing to the decline in the investment and/or high cost of drug development and discovery [137,138], limited large-scale production of antimicrobials from natural sources due to their naturally occurring low concentrations, as well as a lack of innovative and sophisticated drug discovery tools. Traditional approaches to drug discovery from microorganisms include the following:

- Diffusion methods have several types, including agar disk diffusion, antimicrobial gradient, agar well diffusion, agar plug diffusion, cross streak, and poisoned food methods. The agar disk diffusion method is a routine microbial susceptibility test that was developed in 1940 [139]. It is conducted to test for certain fastidious bacterial pathogens, such as *Streptococci*, *Haemophilus influenza*, *Neisseria gonorrhea*, *Nisseria meningitidis*, and *Haemophilus parainfluenza* [140]. In this test, a desired concentration of the test compound is placed on the surface of agar-containing microbes. Antimicrobial agents in the test compound diffuse into the agar and inhibit the proliferation of susceptible microbes. The diameter of inhibition growth zones is then measured [141]. Currently, this method is used to test for non-dermatophyte filamentous fungi using the antifungal disk diffusion approach [142]. Although agar disk diffusion cannot accurately determine the minimal inhibition concentration (MIC), it is simple and less expensive to practice [141].
- The antimicrobial gradient method (Etest) involves a combination of dilution and diffusion methods to determine the MIC value of antibiotics, antifungals, and antimycobacterials. This method can also be used to determine the combined effect of two drugs [141,143]. Other diffusion methods, as mentioned, are agar plug diffusion [144], cross streak [145], and poisoned food [146,147] methods [141].
- The dilution method is suitable for determining MIC values of fastidious or non-fastidious bacteria, yeast, and filamentous fungi [141]. Either broth or agar dilution can be used depending on the test being performed. In testing the action of antifungal drug agents, combinations against *Candida* sp. *Aspergillus*, *Fusarium*, and dermatophytes, agar dilutions are mostly used [148,149,150].
- The time-kill test [151], ATP bioluminescence assay [152,153,154,155], and flow-cytofluorometric method [156] are all techniques used to screen and determine the susceptibility of microbes to antimicrobial compounds [141]. ATP bioluminescence has been used to estimate the amount of ATP present in different cell types [152]. The luciferin–luciferase bioluminescent assay method is mostly preferred due to its high sensitivity [152]. In this method, MgATP2^+^ changes luciferin into a state that can be catalytically oxidized by the luciferase in high quantum yield chemiluminescent reaction [152]. There is a relationship between light intensity and ATP concentration under the right conditions [152]. Cellular ATP can be measured when free ATP released from broken down cell is made to react with the luciferin–luciferase resulting in light emission [152]. The amount of light emitted is measured by a luminometer [141].
- The time-kill test on the other hand, is suitable for evaluating bactericidal and fungicidal activity [141]. It provides information about the relationship between the antimicrobial agent and the microbial strain depending on the time taken for the action to occur and the concentration of the antimicrobial agent [141].
- The flow cytofluorometric method exposes antimicrobial resistance and predicts the effect of the tested molecule on cell damage and viability of the tested microbe [141] using a flow cytometer [157]. In performing this procedure, the cells damaged by antimicrobial agents are dyed with an appropriate stain [141]. A known DNA stain is propidium iodide (PI) [141]. The quantity of damaged cells can be used to determine the antimicrobial activity of the test compound [141].

**Figure 2:**
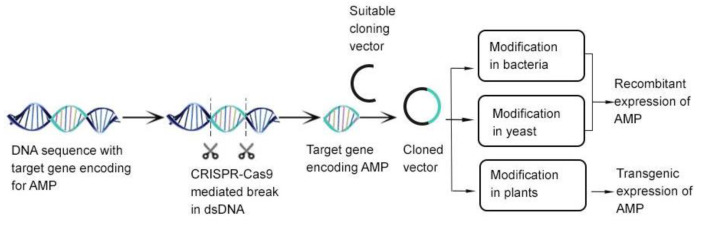
CRISPR-Cas 9 combination with existing gene-editing tools in the discovery of novel AMPS. Image adapted from [195] with some modifications.

However, the availability of the pathogen genomic-scale dataset, modern biomedicine research tools, and the presence of novel model organismal systems has paved the way for bioprospecting of new antimicrobial compounds [158,159,160]. In recent drug development, traditional wet-lab approaches have been substituted by structural bioinformatics, subtractive genomics, and metabolic pathway analyses [161,162,163,164]. Despite the prospects of in silico approaches in drug discovery, the full spectrum of their capabilities has not been explored. Nonetheless, other molecular and genomic technologies have recently seen some success. Target-based drug discovery, in particular, has enhanced the identification of promising therapeutics, including drugs in the management of HIV/AIDS-resistant infections [165], as well as antibacterial inhibitors of peptide deformylase, a metallohydrolase vital in the survival of pathogenic strains, such as *Mycobacterium smegmatis* [166,167,168,169]. Similarly, genomic studies of AFN-A1252, a potent inhibitor of enoyl-ACP reductase (FabI) enzyme in the fatty acid biosynthesis pathway of *Staphylococcus aureus*, *Burkholderia pseudomallei*, and other pathogenic bacteria, has unraveled the FabI as a potential target in drug development, and more specifically, with in vitro and in vivo biological efficacy [170,171]. The emergence of omics technologies, notably genomics, transcriptomics, and proteomics, has fast-tracked the development of bioinformatics tools to identify novel drug targets and lead compounds. The genome mining technique, as it is popularly called, can be used for the detection and analysis of the biosynthetic gene clusters of the chemical compounds and then connect those genes to molecules [172]. The advancement of artificial intelligence (AI) and machine learning (ML) technologies has also offered scientists alternate ammunition towards the fight against antimicrobial resistance [173]. Aside from the development of halicin, an antimicrobial using ML approaches, AI technologies are significant in all stages of drug discovery, ranging from target validation and identification of predictive biomarkers, to analyzing pathological data in the various stages of clinical trials [174,175,176]. The advent of model organisms, such as *Caenorhabditis elegans* [177], zebrafish [178,179,180], *Drosophila melanogaster* [181,182], *Galleria mellonella* [183,184,185], and *Bombyx mori* [186], have been essential in studying human infections and screening, and/or investigating for new antimicrobials [177,178,179,180,181,182,183,184,185,186,187,188,189]. The study of the quorum sensing machinery of *Caenorhabditis elegans* has aided researchers in identifying lead antimicrobial compounds. The identification of *Chomobacterium violaceum*, a *Caenorhabditis elegans* quorum sensing antagonist known to confer survival to the organism by terminating any bactericidal action, validated the hypothesis that compounds that interfere with bacterial quorum sensing could be isolated and developed as a potential antimicrobial [190]. In addition, these model organisms also permit the identification of microbicidal and microstatic lead compounds. A case in point is esculentin and temporin, which were identified as cationic membrane-active antimicrobial peptides with anti-pseudomonas activity following their ability to promote survival of infected Caenorhabditis elegans [189]. Furthermore, the zebrafish remains an essential model organism currently used in modern biology and biomedical research due to their unique properties, including their optically transparent embryo, completely sequenced genome, developmental processes, affordability, and high-throughput drug screening capabilities. As a model system, the zebrafish are utilized to study pathogen-host interaction, model human infectious diseases, and, importantly, enable the cost-effective rapid screening of millions of antimicrobial drug candidates [178,179,180,191]. High-throughput screening coupled with techniques, such as fluorescence resonance energy transfer (FRET) [192], fluorescence polarization (FP) [193], and homogenous time resolved fluorescence (HTRF) [194], to detect and assay active compounds in samples has significantly reduced the time taken to discover new compounds [195]. The advent of gene-editing technologies, such as clustered regularly interspaced short palindromic repeat (CRISPR) associated protein (CRISPR-Cas) has facilitated an easier modification of host and/or target genes to produce potential and cost-effective recombinant cellular products, including AMPs essential in the fight against the growing antimicrobial resistance [195] see (Figure 2).

## 9. Conclusions

The biodiversity of compounds found in bacteria, fungi, and other microorganisms, and their potent effect is significant in the present age of antimicrobial resistance. The numerous compounds highlighted in this review illustrates the importance of these microorganisms and some novel ways in the search for new antimicrobials in the management of various infections. Further exploration of these organisms will widen the search of new antimicrobials and present us with potent antimicrobials, whose mechanism of action may be unparalleled to available antimicrobials. As such, the incidence of antimicrobial resistance can be significantly reduced.

## Figures and Tables

**Table 1:** Antimicrobial activity of chemical compounds isolated from microorganisms.

Microorganism	Chemical Compound	Molecular Class	Antimicrobial Activity	Reference
*Marinactinospora thermotolerans*	Marthiapeptide A	Cyclic peptide	*S. aureus*, *M. luteus*, *B. subtillis*, *B. thuringiensis*	[12]
*Streptomyces scopuliridis*	Desotamide B	Cyclic peptide	*S. aureus*, *S. aureus*	[12]
*Streptomyces drozdowiczii*	Marfomycins A, B, E	Cyclic peptide	*M. luteus*	
*Verrucosispora* spp.	Abyssomicin C	Spirotetronate polyketides	Methicillin-resistant *Staphylococcus aureus*, Vancomycin-resistant *Staphylococcus aureus*	[12]
*Streptomyces* spp.	Lobophorin F	Spirotetronate polyketides	*S. aureus*, *E. feacalis*	[12]
*Streptomyces* spp.	Lobophorin H	Spirotetronate polyketides	*B. subtilis*	[12]
*Streptomyces* sp.	Caboxamycin	Alkaloid	*S. epidermis*, *S. lentus*, *B. subtillis*	[12]
*Streptomyces niveus*	Marfuraquinocin A, D	Sesquiterpene derivative	*S. aureus*, Methicillin-resistant *Staphylococcus aureus*	[12]

**Table 3:** Bacterial sources of antifungal compounds.

Microorganism	Compound(s)	Susceptible Organism(s)	Reference
*A. hibisca*	Pradimicins A, B, C	*Candida* spp. and *Aspergillus* spp.	[58]
*Actinoplanes* spp.	Purpuromycin	*T. mentagrophytes*	[60]
*Micromonospora species ATCC 53803*	Spartanamycin B	*C. albicans*, *A. cladosporium*, and *Cryptococcus* spp.	[61]
*M. neiheumicin*	Neihumicin	*S. cerevisae*	[61]
*Micromonospora species SCC 1792*	Sch 37137	Dermatophytes and *Candida* spp.	[62]
*B. subtilis*	Iturin A and related peptides	Phytopathogens	[60,72]
*Micromonospora species SF-1917*	Dapiramicins A and B	*R. solania*	[63]
*B. cereus*	Azoxybacilin, Bacereutin, Cispentacin, and Mycocerein	Aspergillus spp., Saccharomyces spp, and *C. albicans*	[60]
*B. lichenformis*	Fungicin M-4	*Microsporum canis*, *Mucor* spp., and *Sporothrix* spp.	[73,74]

## Abbreviations

AMP: antimicrobial peptides; DNA: deoxyribonucleic acid; FP: fluorescence polarization; FRET: fluorescence resonance energy transfer; HTRF: homogenous time resolved fluorescence; LAB: lactic acid bacteria; MAN-PTS: mannose phosphotransferase permease; MRSA: methicillin-resistant *Staphylococcus aureus*; MIC: minimal inhibition concentration; AMPs: antimicrobial peptides; AVPs: antiviral peptides; CRISPR: clustered regularly interspaced short palindromic repeat.
